# Collective detection based on visual information in animal groups

**DOI:** 10.1098/rsif.2021.0142

**Published:** 2021-07-07

**Authors:** Jacob D. Davidson, Matthew M. G. Sosna, Colin R. Twomey, Vivek H. Sridhar, Simon P. Leblanc, Iain D. Couzin

**Affiliations:** ^1^ Department of Collective Behaviour, Max Planck Institute of Animal Behavior, Konstanz, Germany; ^2^ Centre for the Advanced Study of Collective Behaviour, University of Konstanz, Konstanz, Germany; ^3^ Department of Biology, University of Konstanz, Konstanz, Germany; ^4^ Department of Ecology and Evolutionary Biology, Princeton University, Princeton, NJ, USA; ^5^ Department of Biology, University of Pennsylvania, Philadelphia, PA, USA; ^6^ Mind Center for Outreach, Research, and Education, University of Pennsylvania, Philadelphia, PA, USA

**Keywords:** collective behaviour, detection, vision

## Abstract

We investigate key principles underlying individual, and collective, visual detection of stimuli, and how this relates to the internal structure of groups. While the individual and collective detection principles are generally applicable, we employ a model experimental system of schooling golden shiner fish (*Notemigonus crysoleucas*) to relate theory directly to empirical data, using computational reconstruction of the visual fields of all individuals. This reveals how the external visual information available to each group member depends on the number of individuals in the group, the position within the group, and the location of the external visually detectable stimulus. We find that in small groups, individuals have detection capability in nearly all directions, while in large groups, occlusion by neighbours causes detection capability to vary with position within the group. To understand the principles that drive detection in groups, we formulate a simple, and generally applicable, model that captures how visual detection properties emerge due to geometric scaling of the space occupied by the group and occlusion caused by neighbours. We employ these insights to discuss principles that extend beyond our specific system, such as how collective detection depends on individual body shape, and the size and structure of the group.

## 1. Introduction

Being part of a group is an effective strategy for avoiding predation threats [1–4] and locating promising resources [5,6]. Enhanced detection of external objects (for example a predator, or a source of food) is a key aspect of being part of a group, with the benefits referred to as the ‘many eyes’ effect [7,8]. The structure within a group influences how individuals interact with one another and the surrounding environment. For example, groups tend to have more individuals and an increased density under heightened predation risk [9–15] (but see [16,17]). An individual’s position within the group can determine both its possible risk to predation [18], as well as the extent of its social interactions [19,20]. Despite the importance of social grouping for gathering information about the external environment [21,22], there has been little quantification of how within-group structure and the size of the group influence the group’s interactions with their environment.

Many species that form coordinated, mobile groups employ vision as a primary modality for mediating social interactions [23–25]. It is important to consider the actual visual sensory information available to each individual in order to make realistic predictions [26]. Visual connectivity among individuals can predict how a social contagion spreads through a group, such as when ‘informed’ individuals detect and move towards a cue associated with food, and are followed by other naive group members [19,27], or when a startle response propagates across a group [14,20]. As groups get larger, occlusion due to neighbours means that individuals differ in the visual information they have available to them. The available visual information determines whether individuals will respond to other group members [19,20], as well as if any individuals in the group will have the ability to detect cryptic stimuli, such as a predator [7,8]. Simulations demonstrate that effects of visual occlusion increase with the number of individuals, and in particular for very large groups, visual occlusion can even drive fluctuations in internal structure [28].

Here, we examine how the visual information available to individuals in a group depends on both the number of group members and on how individuals are positioned within the group (i.e. the group’s internal structure). We first analyse, quantitatively via computational visual field reconstruction [14,19,20], the visual information available to all individuals within groups of golden shiner fish, whose social behaviour is predominantly mediated by vision [20]. The experiments include groups of different numbers of fish, ranging from 10 to 151 in number. We examine how the detection coverage, which is the angular fraction of the external visual area that an individual can see, depends on the number of group members and an individual’s position within the group. To understand the general principles of collective detection, we formulate a simple model that demonstrates how the observed detection abilities of different groups arise from geometric principles. The model generalizes to show how detection scales when a group contains more individuals and we use these results to discuss the implications and generalizations to other animal groups.

## 2. Results

**Figure 1.**
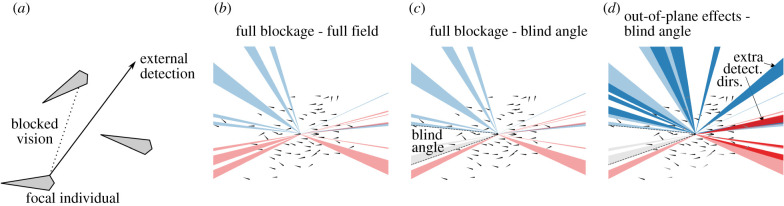
External visual detection of individuals. (*a*) A focal individual has external detection in a given direction if neighbours do not block vision in that direction. Computationally, this is implemented by considering a set of discrete locations outside of the group, which are located far away and thus represent detection in a certain direction (figure 10). (*b*–*d*) External visual detection coverage over all possible directions of a single individual located at the centre of a school of 70 fish. Shown is the detection coverage determined using different parameters. Directions where the left eye has external detection capability are shown in blue, and that for the right eye in red. (*b*) Full visual blockage and a full 360° field of view. (*c*) Including a blind angle where fish cannot see behind themselves, with otherwise full blockage from neighbours. The blind angle area is highlighted by the dotted lines, and detection directions omitted due to the blind angle are shown in grey. (*d*) Blind angle along with out-of-plane effects, where neighbours considered as out-of-plane (shown in grey) do not block the external view in a certain direction. Because tracking is in two dimensions, we approximate this effect by randomly choosing neighbours to designate as out-of-plane, here using a 25% probability that a neighbour will be out-of-plane. Additional detection directions due to out of plane effects are shown in darker colours (compare with *c*).

We filmed free-schooling groups of 10, 30, 70 and 151 golden shiner fish (*Notemigonus crysoleucas*) in the laboratory and used a combination of automated and manual tracking to extract positions and orientations while maintaining individual identities over the course of each trial (see Methods). Golden shiners are a widespread species of freshwater fish [29] that are surface feeders and thus swim close to the surface of the water [30]. We estimated the external visual detection capabilities of each individual using a procedure where a neighbouring individual can block the external vision of a focal individual in a certain direction (figure 1*a*,*b*). This method estimates detection capability by considering visual blockage due to neighbours; thus, the detection capability is a general descriptive measure that reflects the individual and group properties, and is not a representation of a distinct virtual stimulus (see Methods). Individuals tend to have a ‘blind angle’ to the rear, which for this species has been determined to be 25° [31], and we include this in the visual detection procedure (figure 1*c*). In addition, we note while individuals form a relatively planar group structure, near the surface, the arrangement is not perfectly two dimensional. Neighbouring individuals that are not in the same plane may not block detection in a certain direction. Since our tracking is only in two dimensions, we investigate how detection results may be sensitive to out-of-plane effects by using an approximate procedure, where we randomly choose certain neighbours as out of the plane of visual detection, and as therefore not blocking detection in associated directions (figure 1*d*).

**Figure 2.**
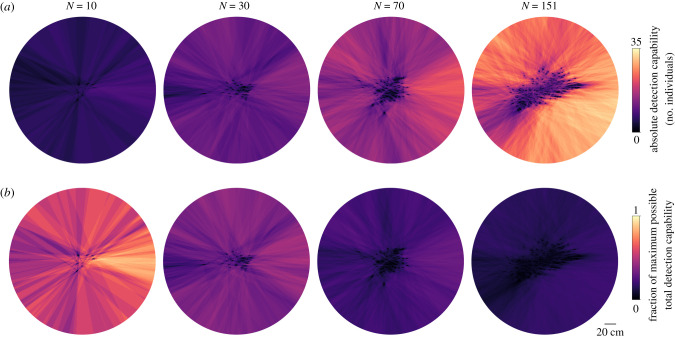
Collective detection capabilities of the group. Illustration of the external visual field of the entire group at a single frame. Each heatmap shows detection capability obtained by summing the overlapping regions of the external visual fields of all individuals, using results with a blind angle and out-of-plane effects (25% out-of-plane probability). Results are displayed by scaling to show either (*a*) absolute detection capability in terms of the number of individuals with detection capability or (*b*) the fraction of the maximum possible total detection capability among group members.

Figure 1 shows examples to illustrate a single individual’s detection coverage outside of the group. Applying the detection algorithm to each individual in the school and overlaying the results illustrates the overall external detection abilities of the group (figure 2) [32].

### 2.1. Individual detection coverage

**Figure 3.**
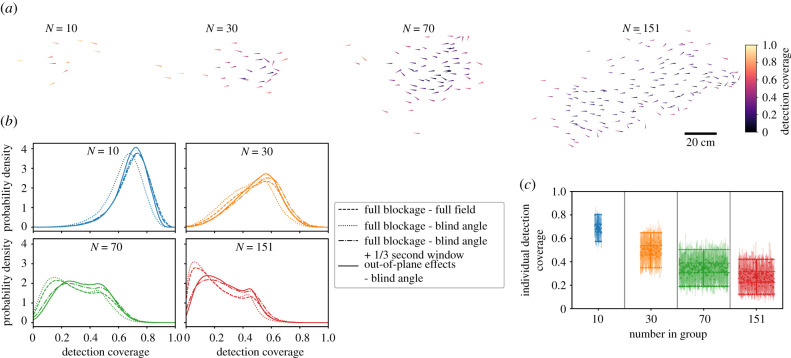
Individual detection coverage. The detection coverage is the fraction of the external visual field that an individual can see. (*a*) Example snapshot of the external detection coverage for groups with different numbers of individuals. (*b*) Distributions of individual detection coverage for the different groups, combining all individuals during a trial, calculated using different settings: full blockage–full field (dashed line), full blockage–blind angle (dotted line), full blockage–blind angle with detection capability any time over a 1/3 second time window (dashed-dotted line), and out-of-plane effects (25% out-of-plane probability)–blind angle. (*c*) Detection coverage, comparing individual differences to the combined distribution. Results use out-of-plane effects (25% out-of-plane probability)–blind angle. The mean and standard deviation of the combined distribution from B are shown as the large point and error bars for each group size. The mean individual detection coverages during a trial are the small points, and individual standard deviations are the shaded bars. Note that 3 trials were performed for *N* = (10, 30, 70), while only 1 trial was performed for *N* = 151. The individual points are spaced on the *x*-axis for display purposes. The dashed line shows the contribution from ‘consistent individual differences’ (the variance of individual means) to the overall distribution, while the dotted line shows the contribution of ‘individuals differing during a trial’ (the mean of individual variances; see Methods, equation (4.5)).

We first examine individual detection coverage, which ranges from 0 to 1 and represents the fraction of the external visual space that an individual can see, and then following this, in §2.2, examine the total number of group members with detection capability in a certain direction at a moment in time. For small groups of 10, all individuals have a large detection coverage and can see nearly the full range around the group, i.e. in directions to the front, back, and side of the group, regardless of their position within the group. As the number in the group increases, however, the average detection coverage decreases due to occlusion caused by neighbours. Additionally, the variance of individual external visual coverage in the group increases with the number of individuals, reflecting an increased heterogeneity in visual access resulting from individuals of the group having their visual field increasingly dominated by others, thus occluding their view of areas external to the group (figure 3). Considering a blind angle decreases the instantaneous detection coverage, with the largest effect for the group of 10. This is because in small groups, the rearward area, in the absence of a blind angle, would be visible, while in large groups, it is likely that vision to the rear is already blocked by a neighbour.

Although we determine detection using the instantaneous positions of individuals in a given frame, actual motion decisions do not occur instantaneously, but rather use information that has been accumulated over a finite amount of time [33]. We therefore ask how small positional changes over a short time interval could affect the detection coverage over time. To represent this, we say an individual has detection capability in a certain direction at time *t* if there was visual access in that direction at any time within the previous *T* seconds, i.e. within the time window of *t* − *T* to *t*. Because this representation uses an OR function to determine coverage (detection capability in a certain direction at time *t*, OR time *t* + 1, etc.), it increases the average detection coverage, with the largest effect on the most numerous group (*N* = 151). For all groups, the results using a blind angle and a time window of *T* = 1/3 s yield average detection coverage values that are near to or greater than that without using a blind angle. This demonstrates that considering small positional changes over a short time could effectively ‘mitigate’ the decrease in detection coverage caused by having a blind angle.

As expected, considering out-of-plane effects increases an individual’s detection coverage, with the largest shift for the most numerous group (*N* = 151). Figure 3 shows results with an out-of plane probability of 25%; this represents the probability that a given neighbour is not in the dominant plane of the group, and therefore does not block external detection. Using other values causes the coverage to progressively increase as the probability of neighbours being out of plane increases. Despite the shifts in distributions when considering a blind angle, time-window averaging, or out-of-plane blockage, we see the same main trends: individual detection coverage decreases and the variance of the distribution of individual detection coverage increases when there are more individuals in the group (figure 3). We do not know precisely how individuals use information over a finite time window, but we do know that real groups do have out-of-plane effects, and that individuals have a blind angle. Because of this, in the following we focus in detail on the instantaneous detection results using a blind angle and out-of-plane effects. While tracking three-dimensional positions could give precise information to relate out-of-plane effects to detection in actual group configurations, figure 3 suggests that general detection trends would be similar, and that an increase in relative out-of-plane positioning predominantly leads to an increase in detection capability on the dominant plane of the group. Although we do not have an exact value of what the effective out-of-plane probability is, we use the intermediate value of 25% as a reasonable value and proceed by focusing on this case, noting that the general features of the results do not depend on the exact value.

The distributions in figure 3*b* combine all individuals over each trial. Are there consistent differences in detection coverage among particular individuals during a trial? We can quantify the contribution of consistent individual differences versus changes over time to the overall variance of detection coverage by calculating the variance of individual mean detection coverage (see Methods, equation (4.5)). This yields that consistent individual differences explain on average only 8% of the total variance, and therefore most of the variance in detection coverage is driven by individuals changing their position within the group over the course of a trial (figure 3*c*).

### 2.2. Collective detection capability

**Figure 4.**
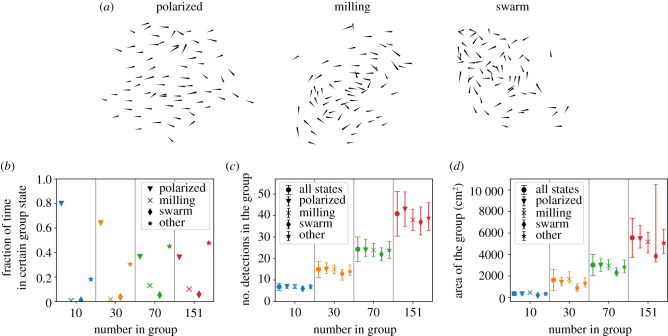
Group states and detection dependence. (*a*) Example snapshots of a group of 70 in different configurations: polarized, milling and swarm states. (*b*) The fraction of time each group was observed in the different states. (*c*) The total number of possible detections for all individuals in the group, for groups with different values of *N*, showing results for all group states compared to polarized, milling, swarm and other states. Points show the median, and error bars show the lower and upper quartiles of the total number of possible detections among all group members in a certain direction at an instant in time. (*d*) The distribution of spatial area occupied by the group, for different states, showing the median (points) and inter-quartile range (error bars). The high upper quartile values for the group of 151 in the swarm state are due to instances where the group is not a single cohesive unit.

**Figure 8.**
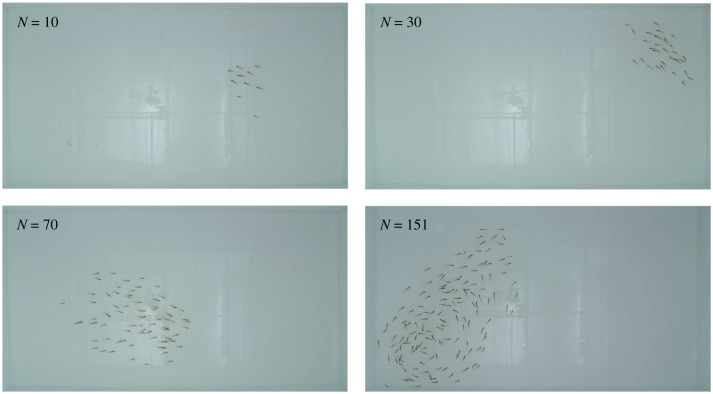
Tank configuration and different numbers of fish. Snapshots showing groups with different numbers of fish in the experimental tank. See also electronic supplementary material video for a short clip, and Data availability to access full videos.

Instead of examining detection coverage of individuals, we can instead ask about the total number of group members with external detection capability in a certain direction at a moment in time (figure 2). This can depend on the group state and group area, e.g. whether the group is swimming in a polarized, milling, swarm, or other configuration (figure 4*a*) [34], as well as on the external direction with respect to the group travel direction. We first examine the former. To define the group states of polarized, milling, swarm or other, we use the group polarization and rotation order parameters, with the same definitions as in [34]. While the polarized state is the most common configuration—the groups of 10 and 30 do not spend significant time in particular in the milling state—the groups of 70 and 151 do spend time in different states (figure 4*b*). The groups of 70 and 151 spend large fractions of time in the other state, which is characterized by intermediate values of the polarization and rotation order coefficients; we note that this may be partially due to boundary and confinement effects which impact this large group more so than the other values of *N* (figure 8). We use the different group states to ask how these configurations impact external detection—see [34] for a detailed analysis of transitions between different collective states and how this is affected by the number of individuals in the group as well as the arena boundary. Here, we see that while the total number of possible external detections among all group members in any direction increases with *N*, it does not strongly depend on the group state (figure 4*c*).

**Figure 5.**
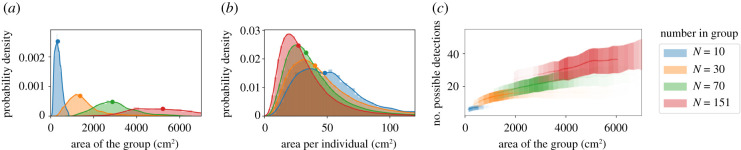
Area occupied and collective detection. (*a*) Distributions of the total spatial area occupied by groups of different numbers of individuals. Points denote the median of the distribution. (*b*) The spatial area per individual, calculated using Voronoi tesselation, for groups of different numbers of individuals. Points denote the median of the distribution. See figure 9 for information on how the total group area and the individual area are calculated. (*c*) The total instantaneous detection capability among all group members, averaged over all possible directions over time, plotted as a function of the total area of the group at that time. The line shows the mean and the shading shows the standard deviation of the number of possible detections. The transparency of the lines is proportional to the probability that the group has a certain area value (see distributions in *a*).

In addition to swimming in different configurations, the density of the group can differ, for example with a dense versus tightly packed group configuration. Naturally, groups with more individuals occupy a larger spatial area. For the different group states, the median area occupied by the group is slightly less in the swarm states compared to other states; for the group of 151, the instances of very large area in the swarm state represent times when the group is not a single cohesive unit (figure 4*d*). The range of values of the spatial area occupied is larger for groups with more individuals (figures 4*d* and 5*a*).

The median spatial area per individual slightly decreases for larger *N*, reflecting that although the distributions were overlapping, individuals tend to pack slightly more tightly when there are more individuals in the group (figure 5*b*). For a group with a given number of individuals, the number of possible external detections is higher when the group occupies a larger area; this is because when individuals are spaced further apart, each neighbour subtends a smaller angle on the visual field of others and therefore blocks less of the external view (figure 5*c*). Overall, these results demonstrate that although detection capability does not depend strongly on group state (figure 4*c*), it has a clear dependence on group area (figure 5*c*).

### 2.3. Angular dependence of detection

**Figure 6.**
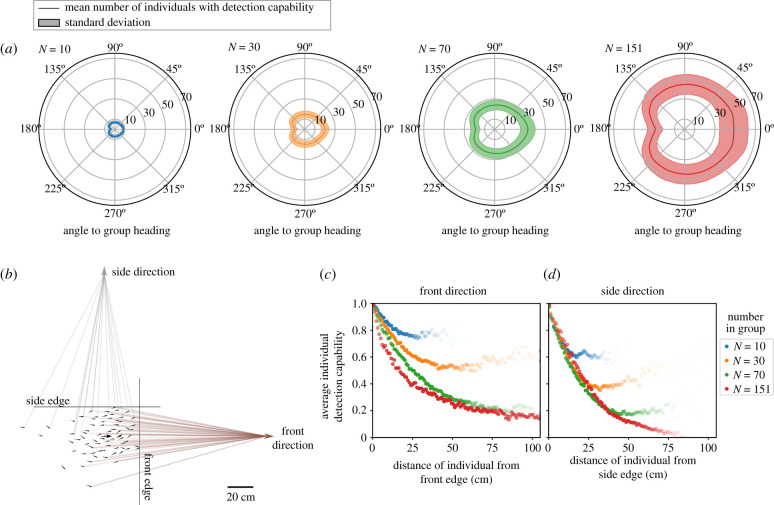
Detection relative to group heading direction. (*a*) The number of possible detections in the group at different angles with respect to the group heading direction. Results are calculated using instances when the group is moving aligned in a polarized state. Lines show the mean number of individuals with detection capability in a certain direction, and shaded area shows the standard deviation. (*b*) Illustration of front and side detection using a snapshot of a group of 70, showing where individuals have open lines of sight to either a location to the front (brown lines) or to the side (grey lines) of the group. The front and side edges of the group are defined by the furthest individual in these respective directions. Computationally, a location far away is used to represent detection in a certain direction (figure 10). (*c*) Average individual detection capability in a direction to the front of the group, plotted as a function of individual distance from the front edge of the group. (*d*) Analogous results to (*c*), but for average individual detection capability in a direction to the side of the group, plotted as a function of individual distance from the corresponding side edge of the group. For (*c*,*d*), the transparency of the points is proportional to the number of observations of individuals at that distance from the front or side.

The number of individuals with detection capability in a certain direction also depends on the angle with respect to the group travel direction. Note that if the group is not moving, then there is no ‘group travel direction’, and no front or back of the group. However, if the group is moving cohesively in a polarized configuration (e.g. figure 4*a*), then there is a clear travel direction and a difference between individuals at the front versus the rear of the group. Because of this, we consider only movement when in a polarized state to examine the angular dependence of detection [34]. For fish, which like many animals have elongated body shapes, detection capabilities are higher to the front of the group than to the side of the group. Due to the blind angle, detection capabilities are lowest to the rear of the group (figure 6*a*) [32].

We next examine how detection capability to the front or the side of the group depends on an individual’s in-group position. In-group position is represented by defining the ‘edge’ of the group as the individual furthest away from the centroid in that direction, and then calculating an individual’s distance from either the front or side edge of the group (figure 6*b*). An individual located at a certain edge always has detection capability in the corresponding direction, and therefore average detection capability is 1 at a distance of zero from the edge. Detection capability then decreases with distance from the edge. However, the decrease in detection capability with distance from the edge depends on both the angle with respect to the group travel direction and the number of individuals in the group. In the smallest group (*N* = 10), nearly all individuals have detection capabilities to the front of the group, and the detection capability shows only a small decrease with distance from the front edge. The steepness of decay of detection capability with distance from the front edge of the group increases with the number of individuals in the group (figure 6*c*).

By contrast, the detection capability with respect to the distance from the side edge of the group shows a similar initial decay for all group sizes, but extends ‘further’ for the larger groups because they take up a larger area (figure 6*d*). This difference between front versus side detection is due to the elongated body shapes of fish as well as the alignment of individuals when swimming as a polarized group. While an individual’s vision to a region to the side of the group may be completely blocked by a single nearby aligned neighbour, visual blockage to a region to the front is more likely to depend on the positions and orientations of several neighbours [32].

Although the presence of tank boundaries has some effect on the distribution of individuals in the group, we expect the same trends regarding differences in front versus side detection (figure 6*c*,*d*) to hold for groups that are moving in an open area. Overall, while we see a weak dependence of detection capability on the group motion state (figure 4*c*), we see a stronger dependence on the overall area occupied by the group (figure 5*c*). This suggests that if the group takes on a different configuration due to the presence or not of a boundary, or if the configuration changes in response to a threat [14], that overall area occupied is a primary driver of collective detection trends, while the specific positioning of individuals has a secondary effect. This agrees with a previous investigation that found that randomizing individual positions within a group to only have a small effect on detection capability [32].

### 2.4. Model of external detection

To understand the general geometric principles that drive collective detection, we formulate a simple model of the external visual detection capability of a group of individuals. Previous work using an agent-based representation found that because individuals change positions over time, the individual properties of body shape and size along with overall group configuration affect inter-individual connectivity and detection more so than the precise relative positions of individuals in the group [32]. Building on this, we represent these aspects as key input parameters—i.e. the probability of visual blockage by a neighbour, and the density of the group—and construct the simplest possible minimal model that is able to capture how visual detection capability changes when there are more individuals in the group. Because of the simplicity of this approach, the model is not specific to our study system of fish, and can be generally used to describe detection capability of any group in a planar configuration.

**Figure 7.**
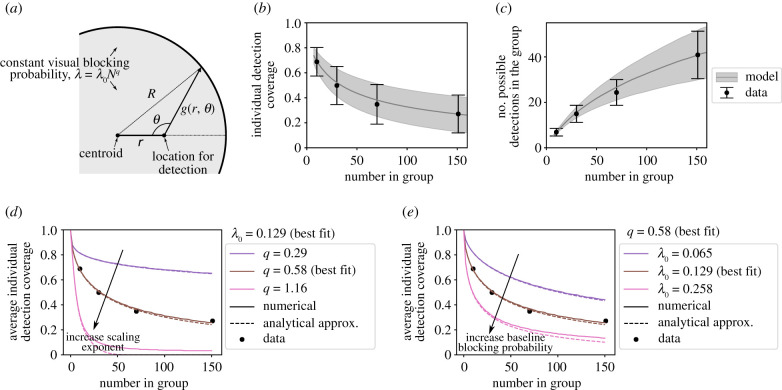
Model of external visual detection coverage. (*a*) Illustration of the quantities in the model. (*b*) Individual detection coverage in the model compared to the data. The baseline blockage probability *λ*_0_ and the scaling exponent *q* are fitted to the average detection coverage in the data, yielding *λ*_0_ = 0.129 and = 0.58. The error bars show the standard deviations of the distributions from the data, and the grey shaded area shows the standard deviation for the model. (*c*) Total number of detections in the group in the model compared to the data. The parameter *σ*, which represents the standard deviation of the radius of the group in the model, is fitted to the standard deviation of the number of possible detections in the data (error bars), leading to *σ* = 0.263. The grey shaded area shows the standard deviation of total detections in the model. (*d*,*e*) Average detection capability for different model parameters and number of individuals in a group. In each, the points show the values from the data, the solid lines are obtained numerically from the model, and the dashed lines are the series approximation in equation (2.2). The solid brown line shows the best fit from model, which is obtained using numerical evaluation. (*d*) Average detection capability for different values of the scaling exponent *q*, with *λ*_0_ set to the best fit value. (*e*) Average detection capability for different values of the baseline blocking probability *λ*_0_, with *q* set to the best fit value. See Methods for model details and fit procedure.

In the model, a group of *N* individuals occupies a circular area with radius *R*, within which there is a constant visual blockage probability of *λ*. At a distance *r* from the centre, the probability of having detection capability at an angle of *θ* is proportional to the blockage probability multiplied by *g*(*r*, *θ*), which is the distance to the edge of the group in that direction (figure 7*a*; see Methods). For a group with *N* individuals, we specify that the visual blocking probability scales according to

2.1 $$\lambda =\lambda_{0}N^{q},$$

where *λ*_0_ is a baseline blocking probability and *q* is a scaling exponent. We fit the values of *λ*_0_ and *q* by comparing individual mean detection probabilities from the model to the data (figure 7*b*).

We furthermore include the effect that a group may change the spatial area it occupies by using a parameter *σ* for the standard deviation of the radius of group. Since a given group occupying a larger area has a higher average number of possible detections (figure 5*c*), spatial area changes increase the standard deviation of the total number of possible detections of group members in a given configuration. We therefore fit the parameter *σ* to the standard deviation of the number of possible detections for the different group sizes (figure 7*c*). Note that all model predictions, for all possible values of *N*, are defined by the three parameters of *λ*_0_, *q* and *σ*.

An approximate solution for the average detection coverage is obtained using a series expansion (see Methods), yielding

2.2 $$\bar{v}\approx e^{-\lambda }\left(1+\sum_{k=1}^{k_{max}}c_{k}\lambda^{k}\right),$$

where $\bar{v}$ is the average detection coverage, *λ* is specified in equation (2.1), and the numerical values of the coefficients *c*_*i*_ are listed in Methods from an expansion to sixth-order terms. Equation (2.2) shows that to leading order, average detection decays exponentially in *λ*. From this, we clearly see that increases in both the scaling exponent (*q*) or the baseline blocking probability (*λ*_0_) both decrease the average detection capability. However, these two parameters affect the shape of the decrease differently, with *q* having more of an effect on the shape of the exponential decay with increasing *N* (figure 7*d,e*).

Overall, the model demonstrates how our experimental findings for detection results are explained by the geometry of how neighbouring individuals occlude an external view; even with fairly drastic simplifying assumptions (a circular shape of the group and a constant blockage probability), the model captures the main experimental trends for how detection scales with *N* (figure 7*b*,*c*). In particular, the model can replicate the experimental observations that average individual detection decreases with *N* (due to an increased probability of occlusion from neighbours), and that the variance of the number of possible detections in the group increases with *N* (due to the variance in the spatial area occupied by the group). Because of the simplicity of the model, we can use it to describe general expected trends of detection for planar groups at different densities, with different scaling properties with *N*, or where individuals have different sizes. A higher value of the baseline blocking probability *λ*_0_ could represent larger individuals, or a higher average density for a given value of *N*. The parameter *σ* represents the standard deviation of the spatial area that the group occupies about the average; since density affects detection coverage, a higher value of *σ* means a higher variance in the number of possible detections in the group. The parameter *q* represents how individual detection coverage changes with the number of individuals in the group (*N*); in particular, a higher value of *q* could represent groups that show a sharper decrease in the area per individual with *N* than we observed experimentally.

## 3. Discussion

In general, in small groups individuals have detection capability in nearly any direction, while in large groups individuals can differ substantially from one another in their visual information due to occlusion from neighbours. For our study system (golden shiner fish), we thus find that meaningful distinctions in available visual information emerge when groups contain between 30 and 70 fish; at these sizes and larger, some individuals may detect an object while others do not. While previous work has used position-based metrics to define, for example, ‘peripheral’ versus ‘central’ locations within a group (e.g. [18,22,35]), we note that these distinctions are only meaningful with respect to detection for groups with sufficiently many individuals.

The density of a group affects visual detection abilities (figure 5*c*). What functional aspects may lead a group to adopt a certain density? Previous work has suggested that up to a certain point, higher group density is associated with an increased ability to spread information among group members; or in other words, denser groups tend to be more ‘tightly connected’ [19,20]. The reason for this, as has been found for animals from fish [20] to humans [36–38], is that the spreading of social behaviour is best described by a fractional contagion process, whereby an individual’s probability of response depends on the fraction of their neighbours that have responded [39]. In denser groups, the closer packing means that close neighbours occupy a large field of view, and therefore individuals will see fewer other group members. Visual access to others was shown to be the best predictor of individual response [19]. In denser groups, each individual has fewer visual neighbours, and thus a contagion is more likely to spread [19,20]. This is further supported by what happens when fish are exposed to schreckstoff, a chemical that is typically released to signal that a predator is nearby. In this case, individuals move closer together, which facilitates an increase in their ability to spread behavioural change, and thus exhibit a greater responsiveness to external threats [14]. While transmission of behavioural change among group members may be enhanced at higher density, our results show that external detection is enhanced at lower density (figure 5*c*). This is because at lower density, neighbours subtend a smaller angle in the visual field of others. In the model, an overall lowering of the density of the group can be represented by a lower value of *λ*_0_. The ‘trade-off’ between external detection and internal communication may be a driver of the optimal group density, and can explain why the overall spatial area of the group does not predict how quickly a group will respond [40]. At an individual level, a low external detection capability to the side of the group tends to be compensated by stronger visual connectivity to neighbours [32].

With more individuals, the overall detection capability of the group increases, due to both having a full coverage of the surrounding area as well as having multiple overlapping visual areas for detection redundancy. However, blockage effects cause this trend to be sub-linear with respect to the number of individuals in the group (figure 7*c*). This demonstrates that one of the benefits of being part of a group—the ‘many eyes’ effect [7,8]—has a decreasing marginal utility as group size continues to increase. To explore possible functional consequences of this, consider that individuals in a group need to not only detect an object, but also respond to the detection. While a predator may elicit a sudden startle response [20], movement towards a potential food source is more likely to be gradual. Previous work has shown that only a small fraction of ‘informed’ group members (e.g. group members that can detect the food source) are needed in order to successfully guide the group towards the target [19,27,41]. Here, we note that although the fraction of informed individuals needed to lead the group decreases with *N*, the average detection capability of each individual also decreases with *N*. Therefore, we can not generalize to say whether small or large groups are expected to have a better ability to both detect and move towards a promising food source, since the scaling of detection capability with *N* depends on the characteristics of individuals and the configuration of the group.

In our calculations, we considered that an individual can detect an outside point in a given direction if they have a clear view in that direction. However, this does not take into account differences in detection capability for near versus far away objects that arise due to visual projection, angular differences when the object is close to the group, and contrast effects. Real objects have a finite size and thus the total angle subtended by the object decreases with distance. An object located close to the group thus projects onto a larger range of angular directions compared to the same object located farther away. This naturally results in a lower overall detection capability if an object is located farther away. For distant objects, the relative angular position of the object with respect to each individual is nearly the same for all group members, regardless of their position within the group. Position within the group, however, strongly affects an individual’s relative angular position to a nearby object. For example, for a group swimming in a polarized configuration, an object located at 90° with respect to the group travel direction will be located greater than $90^{\circ }$ from the travel directions of individuals at the front of the group, and less than $90^{\circ }$ with respect to the travel directions of individuals at the rear of the group. While these angular differences are negligible for objects located far away from the group, they will be significant when the object is located only several centimetres away from the front, back, or side of the group. While such finite group size effects impact the quantitative values of the visual detection capability, they do not affect the general detection trends we analysed in this study, because the general trends are driven by visual blockage due to neighbours. Specific relationships that could be important to certain experiments, such as contrast effects, finite group size effects, and decreases in visibility due to turbidity, could be examined in the relevant context using extensions of our method to estimate detection capability. Nonetheless, since a basic driving factor in detection is the capability to see outside the group, the same qualitative trends with respect to how individual detection scales with group size (figures 3 and 7), depends on group configuration and spatial area (figures 4 and 5), and depends on within-group position and object location (figure 6), are applicable in any context where individuals move in groups in planar configuration.

The contrast an object appears at with respect to the background decreases with distance due to the effects of light scattering and absorption. This can have a significant effect in attenuating media such as water, and can be particularly strong in conditions of poor visibility (e.g. in turbid, or ‘cloudy’, water—see [42,43]). A decrease in contrast with distance could have two effects on detection ability. First, it would lower the effective detection capability for each individual in the group. In the model, this is represented by increasing the effective baseline visual blocking probability *λ*_0_ (figure 7*e*). Second, because visual detection only occurs if an object appears above a certain contrast threshold [43], individuals may be able to detect an object if they are close to it (i.e. located on the side of the group where the object is located), but individuals on the other side of the group may not have sufficient contrast to detect the object. Since such mechanisms represent individuals as imperfect sensors, they affect the many eyes abilities of the group: while a group with a small number of individuals could be certain to detect an object in a condition of clear visibility, the same group may not have any individuals that detect the object in conditions of poor visibility. In a group with a larger number of individuals, the pure increase in numbers makes it more likely at least some individuals are able to detect an object even in conditions of poor visibility. This is similar to the ‘pool-of-competence’ effect, whereby larger groups effectively act as better problem solvers because it is more likely they contain an individual that has the knowledge needed to solve the problem [44,45]. Applying this to the case of visual detection, a larger group is more likely to contain an individual that can detect the object.

While we obtained data from freely moving groups of fish, we note that the effective transition point from homogeneous to heterogeneous visual information available among individuals will be different for groups of different animals. Based on our results, we would expect differences due to the shape of the animal, the spacing between individuals in the group, and the overall space that the group occupies. For example, while we studied fish moving in a planar configuration in shallow water, and approximated out-of-plane effects using probabilistic visual blockage, we expect that fish moving in a fully three-dimensional (non-planar) shape would have overall a smaller fraction of their vision blocked by neighbours, for a group containing the same number of individuals. However, experiments also show that fish schools in open water often adopt planar structures, in particular in response to a nearby predator [46], and that using two-dimensional motion coordinates yields the same results for leader–follower dynamics as considering full three-dimensional motion [47]. Other animals that form non-planar groups [48], such as midges or birds, can differ in the effective visual blockage due to neighbours. In a midge swarm, where the inter-individual spacing relative to body size is larger than that for fish [49], we would expect relatively low visual blockage. Different from fish, we would also expect minimal directional dependence, due to the body shape of midges. In contrast to midges, birds have elongated body shapes, and therefore we could expect similar direction-dependent detection trends for birds as we found for the fish schools studied here; in addition, although birds move in three dimensions, data from starlings have shown that flocks are generally thinnest in the direction of gravity and therefore also have planar characteristics [50]. Ungulates moving in a herd, such as zebra, gazelles, caribou or wildebeest (e.g. [51,52]), have both elongated body shapes and move on a two-dimensional surface, and thus may have directly comparable trends for visual detection as fish moving in shallow water.

We used a minimal model to show how individual and collective detection capabilities scale with the number of individuals in the group. Because of the simplicity in our model, we expect it to describe the general scaling trends of detection for any group in a planar configuration for the different animal groups in all of these cases. However, our modelling approach does not capture specific differences, that for example may be due to the shape of the group (e.g. elongated or more circular groups), or direction dependencies related to the shape of individuals (e.g. the differences in front versus side detection seen in figure 6). As a complementary approach, agent-based modelling can be used to understand specific differences due to such effects (e.g. [32]).

In summary, we used fish as a model system to examine the visual information available to individuals in the group, and formulated a simple model to show how visual information changes with number of individuals in the group. In future work, it will be valuable to compare results to other animal groups that vary in their individual properties and group dynamics, and to test the expected changes in detection ability with respect to individual placement and group motion direction.

## 4. Methods

### 4.1. Experiments

Golden shiners (*Notemigonus crysoleucas*) are a small minnow native to the northeastern USA and Canada [29]. Juvenile shiners approximately 5 cm in length were purchased from Anderson Farms (www.andersonminnows.com) and were allowed to acclimate to the laboratory for two months prior to experiments. Fish were stored in seven 20-gallon tanks at a density of approximately 150 fish per tank. Tank water was conditioned, de-chlorinated, oxygenated, and filtered continuously. Fifty per cent of tank water was exchanged twice per week. Nitrates, nitrites, pH, saline and ammonia levels were tested weekly. The room temperature was controlled at 16°C, with 12 h of light and 12 h of dark, using dawn–dusk simulating lights. Fish were fed three times daily with crushed flake food and experiments were conducted 2–4 h after feeding. These methodologies are identical to those used in [53].

Trials with groups of 10, 30 and 70 shiners (3 trials each) and with 151 shiners (1 trial) were allowed to swim freely in a 2.1 × 1.2 m experimental tank. Water depth was 4.5–5 cm. Fish were filmed for 2 h from a Sony EX-1 camera placed 2 m above the tank, filming at 30 frames per second (figure 8).

The arena was acoustically and visually isolated from external stimuli: two layers of sound insulation were placed under the tank, and the tank was enclosed in a tent of featureless white sheets. Trials took place in a quiet laboratory with no people present during filming. All experimental procedures were approved by the Princeton University Institutional Animal Care and Use Committee.

### 4.2. Tracking and group area

We focused analysis on a 13 min segment for each trial. We chose a time 1 h after the onset of the trial to minimize stress on the fish from handling. Fish positions, orientations and body postures were extracted from videos via the SchoolTracker algorithm used in [20]. Briefly, SchoolTracker works by detecting fish in each frame, then creating tracks by linking detected fish across frames. We then performed manual data correction to ensure accuracy in the tracks.

**Figure 9.**
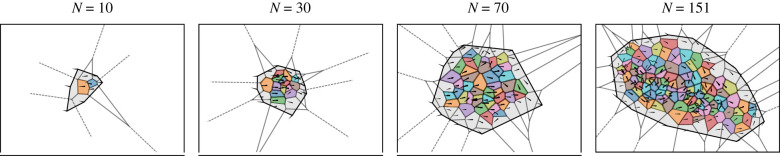
Group and individual area calculations. (*a*) The area of the group is calculated by a convex hull that contains the head positions of all group members (grey shading). Individual area is calculated using a Voronoi tesselation, keeping only Voronoi polygons that are enclosed in the overall group boundaries (coloured areas).

We used a convex hull and Voronoi tessalation to quantify the overall spatial area occupied by the group as well as the spatial area per individual (figure 9).

### 4.3. External detection

**Figure 10.**
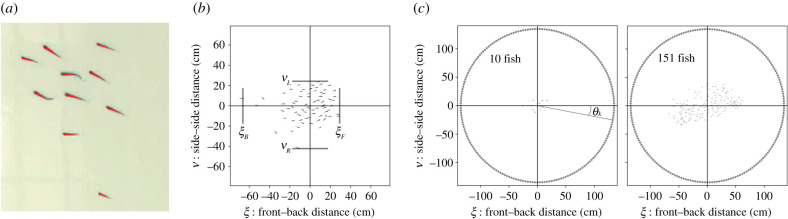
Polygon representation of fish and detection analysis quantities. (*a*) Example zoomed-in video frame from a group of 10 fish with the four-sided fish polygon model shown as the red overlay. (*b*) By setting the origin at the group centroid and the group travel direction along the *x*-axis, we define the (*ξ*, *ν*) coordinate system. The front–back coordinate is *ξ*, and the side–side coordinate is *ν*. The front, back, left side and right side of the group (*ξ*_*F*_, *ξ*_*B*_, *ν*_*L*_ and *ν*_*R*_, respectively) are defined as the head position of the individual farthest away from the group centroid in that direction. The group direction of travel is along the *x*-axis. (*c*) To examine detection, we consider *m* points placed at a distance of *L* from the group centroid; we used values of *m* = 200 and L=1200 pixels (135 cm), and note that none of the results depend on these exact values; we use this representation for simplicity to represent detection with respect to different locations. The angle *θ*_*k*_ defines the angular location of an external point with respect to the group travel direction.

To examine external detection, we represent individuals as simplified four-sided polygons defined by their head, eyes, and tail (figures 1*a* and 10*a*). We compute detection capability using an algorithm that considers *m* points located in a circular arrangement at a distance *L* from the group centroid. Note that geometry dictates that individuals will have different relative angles with respect to a point located close to the group (i.e. small *L*), but all group members have the same relative angle to an point located at an infinite distance away (i.e. for large *L*). In order to obtain dominant trends and simplify our analysis, we use a large value of *L* such that results do not depend on the precise value. With this, the results represent an angular dependence of detection, and not detection of a discrete virtual stimulus; thus *L* should be seen as a parameter that enables a simple computational algorithm to yield a well-defined estimate of visual detection capability. We use this representation because we wish to analyse general trends in detection capability, which are driven by visual blockage due to the presence of neighbouring individuals. We note that the same algorithm for estimating detection capability could be used to examine quantitative differences in detection capability due to finite size effects if a particular experiment was to consider distinct stimuli at a specified distance *L*. We use the values *m* = 200 and L=1200 pixels (135 cm), noting that none of the results depend on these exact values. In our representation, we specify that an individual has direction capability in a certain direction if there is a clear visual line in that direction, without blockage from neighbours (figure 1*a*).

To determine external detection coverage, at each time step, we first shift coordinates such that the group centroid is at the origin, and follow this by a rotation that sets the direction of travel of the group centroid to be along the *x*-axis. In this coordinate system, each individual’s location is defined by a front–back distance *ξ*_*i*_(*t*) along the *x*-axis, and a side–side distance *ν*_*i*_(*t*) along the *y*-axis (figure 10*a*). The edge of the group in each direction is defined as the individual furthest away in that direction; we denote these values as *ξ*_*F*_(*t*), *ξ*_*B*_(*t*), *ν*_*L*_(*t*) and *ν*_*R*_(*t*) for the front, back, left and right edges, respectively (figure 10*a*). In the (*ξ*, *ν*) coordinate system, we expect side-to-side symmetry for reflections about the *ξ*-axis. However, due to both eye positions being located at the head, and a ‘blind angle’ where individuals cannot see behind themselves, there is no front–back symmetry. We used a blind angle value of 25°, which was obtained from a visual study of our study species [31].

Figure 10*b* shows both a small and large group with the ‘circle of points’ surrounding it. Each point has an angular location *θ*_*k*_ relative to the direction of travel of the group centroid, where *k* = 1… 200. Individual *i* has both left and right eyes located to the sides of its body, the positions of which were estimated from the tracking software. We say that individual *i* has detection capability at relative angle *θ*_*k*_ at time *t* if there is no visual blockage between either its left eye or its right eye and the point at *θ*_*k*_. This defines the function

4.1 $$h_{tik}^{\left(N\right)}=\left\{ \begin{array}{ll} 1,\; & \mathrm{at}\;\mathrm{time}\;t,\;\mathrm{individual}\;i\;\mathrm{can}\;\mathrm{detect}\;\mathrm{the}\;\mathrm{point}\;\mathrm{at}\;\theta_{k}\\ 0,\; & \mathrm{otherwise},\; \end{array}\right.\;$$

which is calculated for each group containing a different number (*N*) of individuals.

To approximate out-of-plane effects, we use an analogous calculation to that described above, but instead define a probability that the presence of a neighbour in a certain direction blocks external vision in that direction. This is done with consistent random draws that affect the left eye and right eye together. To consider the blind angle to the rear of an individual, we simply exclude directions within the blind angle and mark them as not detected. Although other than the blind angle we did not place an explicit limit on the range of the left eye versus the right eye in the detection calculations, an individual’s own body blocks vision to their opposite side, so that the left eye does not have a clear visual path to the right side, and vice versa.

We use equation (4.1) to calculate the distributions of individual detection coverage and the total number of detections in the group. Using 〈· 〉 to represent an average over the specified indices, first we define the following notation to simplify the calculations of individual detection:

4.2 $$h_{ti}^{\left(N\right)}=\langle h_{tik}^{\left(N\right)}\rangle_{k},$$

which is the individual detection at an instant in time, calculated by averaging over all possible detection directions *k*. The average individual detection coverage is

4.3 $$H_{\mathrm{indiv}}^{\left(N\right)}=\langle h_{tik}^{\left(N\right)}\rangle_{t,i,k}=\langle h_{ti}^{\left(N\right)}\rangle_{t,i}.$$

The variance of individual detection coverage is

4.4 $${\left(\Delta H_{\mathrm{indiv}}^{\left(N\right)}\right)}^{2}=\langle {\left(h_{ti}^{\left(N\right)}-H_{\mathrm{indiv}}^{\left(N\right)}\right)}^{2}\rangle_{t,i}$$

4.5 $$=\underset{\mathrm{mean}\;\mathrm{of}\;\mathrm{individual}\;\mathrm{variances}}{\underbrace{\langle {\left(h_{ti}^{\left(N\right)}-\langle h_{ti}^{\left(N\right)}\rangle_{t}\right)}^{2}\rangle_{t,i}}}+\underset{\mathrm{variance}\;\mathrm{of}\;\mathrm{individual}\;\mathrm{means}}{\underbrace{\langle {\left(\langle h_{ti}^{\left(N\right)}\rangle_{t}-H_{\mathrm{indiv}}^{\left(N\right)}\right)}^{2}\rangle_{i}}},$$

where the second line follows because the individual and temporal differences are symmetric about the mean. The first term in equation (4.5) is the mean of the individual variances, which is associated with individuals having different values of detection coverage during the course of a trial. The second term in equation (4.5) is the variance of the individual means, which is associated with consistent individual differences. Applying this to the data with results from out-of-plane effects (25% out-of-plane probability) and blind angle, we obtain that the variance of the individual means explains (8.2%, 7.7%, 6.4%, 9.0%) of the total variance for the group sizes of *N* = (10, 30, 70, 151), respectively, with the remaining fraction of the variance accounted for by the mean of the individual variances.

The average number of detections in the group is

4.6 $$H_{\mathrm{group}}^{\left(N\right)}=\langle \sum_{i=1}^{N}h_{tik}^{\left(N\right)}\rangle_{t,k}=N\langle h_{tik}^{\left(N\right)}\rangle_{t,i,k}.$$

Note that while the averages in equations (4.3) and (4.6) are related by the simple formula $H_{\mathrm{group}}^{\left(N\right)}=NH_{\mathrm{indiv}}^{\left(N\right)}$, the full distributions of individual and total detections in the group do not have such a simple relation to each other. The standard deviation of the number of detections in the group is

4.7 $$\Delta H_{\mathrm{group}}^{\left(N\right)}=\sqrt{\langle {\left(\sum_{i=1}^{N}h_{tik}^{\left(N\right)}-H_{\mathrm{group}}^{\left(N\right)}\right)}^{2}\rangle_{t,k}},$$

which is shown in figure 7*c*.

To compute detection with respect to the group direction of travel in figure 6, we use only polarized group states where the direction of travel is well defined. To categorize when the group is in a polarized state or the other states used in figure 4, we calculate the group’s polarization and rotation order parameters using definitions and threshold values to define the different states following [34].

### 4.4. Model

We formulate a simple model to describe the external visual detection coverage of individuals in a group. In this model, the group occupies a circular area with radius *R*, within which there is a constant visual blockage probability. Using symmetry, an individual’s field of view depends solely on its distance *r* from the centre of group, where 0 ≤ *r* ≤ *R*. Whether or not an individual located at *r* can see outside the group in a direction *θ* depends on the distance *g*(*r*, *θ*) from the individual to the edge of the group in that direction (figure 7*a*). Using the law of cosines, this distance is

4.8 $$g(r,\theta )=r\cos\theta +\sqrt{R^{2}-r^{2}\sin^{2}\theta }.$$

We say that the probability of being able to see outside the group in a given direction is the product of the blockage probability *λ* times the distance to the edge in that direction. Assuming that blocking events are randomly distributed and occur with a uniform probability through the group, we use the Poisson distribution to represent the probability of external detection:

$$P_{\mathrm{ext}}(r,\theta )=e^{-\lambda g(r,\theta )}.$$

For the individual at position *r*, the total external detection capability is an average, calculated by the integral over all possible angles:

4.9 $$v(r)=\frac{1}{2\pi }\int_{-\pi }^{\pi }\;e^{-\lambda g(r,\theta )}\;d\theta .$$

To perform calculations, we set *R* = 1, which is done without loss of generality because detection in equation (4.9) depends on the product in the exponent.

Thus far we have assumed that the group occupies a fixed area defined by the radius *R*. However, in the data we observe that groups change the area they occupy over the course of a trial (figure 5). To represent a distribution of the area occupied, consider a group at two different sizes: *R* (the average radius), and *R*_1_ (the ‘current’ radius). Defining the ratio *α* = *R*/*R*_1_, the distance to the edge of the group scales as *g*_1_(*r*_1_, *θ*) = *g*(*r*, *θ*)/*α*. For the blockage probability, we expect this to scale with the density within the group, and thus have *λ*_1_ = *λα*^2^. For a current configuration of the group defined by the size ratio *α*, the external visual detection coverage of an individual is calculated by using equation (4.9) in the current configuration:

4.10 $$v(r,\alpha )=\frac{1}{2\pi }\int_{-\pi }^{\pi }e^{-\lambda_{1}g_{1}(r_{1},\theta )}\;d\theta =\frac{1}{2\pi }\int_{-\pi }^{\pi }\;e^{-\lambda g(r,\theta )\alpha }\;d\theta .$$

For simplicity, we represent different group areas by using a Gaussian with a mean of *α* = 1 to represent different possible values of the group radius,

4.11 $$P(\alpha )=\frac{1}{M}\exp\left(-\frac{{\left(\alpha -1\right)}^{2}}{2\sigma^{2}}\right),$$

where *σ* represents the magnitude of changes in the group radius, and *M* is a normalization factor. Because the radius must be positive, we restrict to values *α* > 0.

To compute the probability distribution of external detection, we evaluate equation (4.10) on a discrete set of radii, calculating the number of individuals in a shell around a given value of *r* as proportional to

4.12 $$n(r,\alpha )=\pi ({\left(r+\delta \right)}^{2}-r^{2})P(\alpha ),$$

where *δ* is the width of the shell. We then use binning to calculate the probability distribution of detection coverage, using equation (4.11) to represent the probability of different group areas. To apply the model to the groups with different numbers of individuals, we specify that *λ* varies to a power of the number of individuals (*N*) in the group (main text equation (2.1), repeated here):

$$\lambda (N)=\lambda_{0}N^{q}.$$

The model results for all values of *N* are defined by the three parameters *λ*_0_, *q*, and *σ*. Because the average detection coverage depends only very weakly on the value of *σ*, we use a two-step procedure to fit these parameters to the data, fitting *λ*_0_ and *q* to the average detection coverage, and then subsequently fitting *σ* to the standard deviation of the total number of detections in the group.

Note that individuals maintaining constant density with an increase in *N* can be approximated with *q* = 0.5 (this represents a linear increase in area with *N*). However, even if individuals did maintain constant density, this scaling would only be strictly true for point particles. To see why, consider the case where individuals are zero-dimensional ‘points’; then, the visual blockage probability would only depend on the density of points, and would be constant with distance for uniformly distributed points. However, since instead a group member has a two-dimensional projection represented in our calculations by a polygon, the visual blockage probability depends both on the density of neighbours and the distance from each observer. Because of this, we fit both the values of *λ*_0_ and *q*. The fitting procedure for these parameters minimizes the mean square error of the model result for mean external detection capability compared to the data for each value of *N*, where values from the data are used that consider out-of-plane effects with a 25% out-of-plane probability (figure 7*b*).

Following this, we fit *σ* by minimizing the mean square error of the model result for the standard deviation of the total number of detections in the group compared to the data (figure 7*c*). Note that in the model, a single configuration of the group is defined by a particular value of the group area.

#### 4.4.1. Analytical approximation for average detection capability

To obtain an analytical approximation of mean model detection results, consider equation (4.9), which is the detection capability of an individual located at position *r*. Using a single value for the group area, the average visual degree is an integral over the unit sphere of equation (4.9) times the probability that an individual is located at *r*:

4.13 $$\bar{v}=\frac{1}{\pi }\int_{-\pi }^{\pi }\int_{0}^{1}\;e^{-\lambda \left(r\cos\theta +\sqrt{1-r^{2}\sin^{2}\theta }\right)}r\;d\theta \;dr.$$

Although this cannot be evaluated in closed form, we can obtain an approximation by considering the series expansion in powers of *r*:

4.14 $$\bar{v}=\frac{1}{\pi }\int_{-\pi }^{\pi }\int_{0}^{1}\;e^{-\lambda }\left(r-\lambda \cos\theta r^{2}+\frac{1}{2}\lambda (\lambda \cos^{2}\theta +\sin^{2}\theta )r^{3}+\cdots \right)d\theta dr.$$

Evaluating the integral for the individual terms yields an expression in the form of an exponential times a series expansion in powers of *λ* (main text equation (2.2), repeated here):

$$\bar{v}\approx e^{-\lambda }\left(1+\sum_{\;j=1}^{\;j_{\max}}c_{\;j}\lambda^{\;j}\right).$$

Keeping terms to sixth order in *r* has *j*_max_ = 6 and the following coefficient values: *c*_1_ = 0.1455, *c*_2_ = 0.1455, *c*_3_ = 1.302 × 10^−2^, *c*_4_ = 6.185 × 10^−3^, *c*_5_ = 3.255 × 10^−4^, *c*_6_ = 1.085 × 10^−4^. We use the above expression (equation (2.2)) with these coefficient values to plot the series approximation in figure 7*d*,*e*.

## Data accessibility

Data are available at https://datadryad.org/stash/share/UH5t3WTf-PJPjzxmgDk1V2B7HwcsJiz1Nk6UVrSuO6A, and code is available at https://github.com/jacobdavidson/collectivedetection.
